# Characterizing the interaction between physicians, pharmacists and pharmaceutical representatives in a middle-income country: A qualitative study

**DOI:** 10.1371/journal.pone.0184662

**Published:** 2017-09-12

**Authors:** Rima Hajjar, Aya Bassatne, Mohamad Ali Cheaito, Rabie Naser El Dine, Sarah Traboulsy, Fadi Haddadin, Gladys Honein-AbouHaidar, Elie A. Akl

**Affiliations:** 1 Faculty of Medicine, American University of Beirut, Beirut, Lebanon; 2 Hariri School of Medicine, American University of Beirut, Beirut, Lebanon; Jagiellonian University, POLAND

## Abstract

**Background:**

Studies around the world have shown that interactions between pharmaceutical companies, pharmacists and physicians have a great influence on prescribing and drug dispensing practices. In middle-income countries, the nature and extent of these interactions have not been well researched. Our objectives were to qualitatively explore the nature of the interactions between pharmaceutical companies, physicians and pharmacists, their impact on drug prescription and dispensing practices in Lebanon.

**Methods and findings:**

We used grounded theory approach as well as the known sponsor, purposive, and snowballing sampling strategies to identify interviewees from the three respective groups: physicians, pharmacists, and pharmaceutical representatives. We conducted semi-structured and analyzed transcripts thematically. This study encompassed 6 pharmaceutical representatives, 13 physicians and 13 pharmacists. The following themes emerged: purpose and driver for the interactions, nature of the interactions, incentives, impact on prescription practices, ethical considerations, and suggestions for managing the interactions. The main purposes for the interaction were educational, promotional, and monitoring prescription practices and dispensing, while the main drivers for these interactions were market potential and neighborhood socio-economic status. Physicians, pharmacists and pharmaceutical representatives who engage in these interactions benefit from a variety of incentives, some of which were characterized as unethical. It appears that pharmaceutical companies give prominence to selected physicians within their communities. Although members of the three interviewed groups refer to some of the interactions as being problematic, they described a culture of acceptance of gift giving. We developed a framework that depicts the prevailing politico-cultural environment, the interactions between the three professional groups, and their impact on drug prescription. Underreporting is the main limitation of this study.

**Conclusion:**

Interactions between physicians, pharmacists and pharmaceutical representatives are frequent. Although these interactions can be beneficial, they still have a substantial effect on drug prescription and dispensing practices. Hence, the need for new policies that regulate these interactions and penalize any misconduct.

## Introduction

Pharmaceutical companies allocate a significant annual budget for marketing their products. Part of this budget is allocated to direct-to-consumer advertising [1], with national television and magazines being the favored media platforms for pharmaceutical advertisers. Another part is allocated to physicians and pharmacists in the form of medical education activities such as symposia, direct monetary benefits, travel funds, and research grants [1, 2].

An analysis of the 2014 data of the Open Payments program found that 1,444 pharmaceutical companies reported paying 6.5 billion USD to more than 600,000 physicians and 1,100 teaching hospitals in the United States [3]. Physicians and pharmacists’ engagement in promotional activity offered by pharmaceutical company is a global phenomenon. An estimated 65%, 69% and 99.5% of physicians in US, Canada and Saudi Arabia respectively receive pharmaceutical industry payment [4, 5] [6].

Several studies have explored the nature of marketing strategies by pharmaceutical companies in various high-income countries, such as United States and Canada [1, 3, 5, 7]. DeAngelis describes a heavily incentive-laden strategy targeting various medical professionals. Medical and pharmacy students are incented with books, free seminars and lunches; physicians and pharmacists are showered tickets to sporting and other events; and academic physicians and pharmacists are sponsored, by pharmaceutical companies, to publish research [7].

A recent systematic review identified moderate quality evidence for the association between physicians' interactions with pharmaceutical companies and their prescribing patterns and quality[8]. Another systematic review examined the beliefs and attitudes of physicians towards those interactions [9]. One of the major findings was that physicians believe that such interactions have a minor impact on their prescription behavior. Moreover, physicians perceive such impact to be lesser when asked about their own behavior[10]. Therefore, it seems that the impact of these interactions on prescribing behavior often escapes the notice of physicians.

Many high-income countries have enacted laws to control the interaction between pharmaceutical companies and the medical profession. The United Kingdom (UK) introduced the UK bribery Act in 2009 to penalize offences related to bribery. In the US, the Sunshine Act, also known as section 6002 of the Affordable Care Act (ACA) of 2010, forces pharmaceutical companies to post almost all of their payments to physicians on a searchable government website under the recipient's name [11]. Similarly, most multinational pharmaceutical companies have adopted ethical codes of conduct to regulate their interaction with medical professions, “prevent or discourage potentially unethical practices” and “assist both medical practitioners and representatives to conform to an agreed standard of promotional activities” [12].

Lebanon is an upper middle-income country, with about 40% of the population having no health insurance coverage. Although only around 20% of drugs consumed are thought to be reimbursed, Lebanon has one of the highest per capita pharmaceutical spending in the Middle Eastern region[13]. Medications expenses consumed over 25% of household annual expenditures in 2009 [14], compared to an average of 17% in countries of the Organization for Economic Co-operation and Development (OECD) [15].

The aims of this study were to qualitatively explore the nature and intensity of the interaction between pharmaceutical representatives, physicians and pharmacists, and their impact on drug prescription and on dispensing practices in Lebanon. The specific research questions were to:

1. Describe the purpose of the interactions, their nature, and their frequencies;
2. Identify the types of incentives offered;
3. Explore the differential interactions with national and international pharmaceutical companies;
4. Assess the perceived impact of those encounters on prescription practices
5. Characterize the prescription monitoring strategies adopted by pharmaceutical companies.

## Methods

### Study design

We opted for a qualitative design to allow an in-depth exploration of a complex situation without missing out on any subtleties [16]. In fact, none of the questionnaires we had identified to run a quantitative survey addressed the issues we learned about as we were preparing for our project. The qualitative design allowed us to document such issues, including some types of incentives and monitoring strategies used.

We followed the Grounded Theory (GT) methodology based on Corbin and Strauss[16]. We used the systematic qualitative approach of GT for collecting data, analyzing and synthesizing constructs with the intent of inferring a middle range theory. This theory was meant to provide one framework linking between the important determinants governing the interaction, the nature of the relationships between the different players, and the eventual impact of those determinants on prescription practices.

We followed the consolidated criteria for reporting qualitative research (COREQ) in drafting this manuscript [17]. COREQ guidelines include a 32-item checklist that can help researchers to report important aspects of the research team, study methods, context of the study, findings, analysis and interpretations (S1 File).

### The research team

The research team consisted of eight members. Six team members (RH, AB, MC, ST, RN, FH) were second year medical students and had no previous experience with qualitative research. Two team members (GHA and EAA) had repeated experience in conducting and publishing qualitative studies.

### Participants

Our three target populations consisted of physicians, pharmacists, and pharmaceutical representatives practicing in Lebanon.

The corresponding sampling frames were:

1. Physicians practicing in clinics and/or hospitals in Lebanon;
2. Pharmacy staff, including pharmacists and pharmacy technicians, who handle drug prescriptions in an outpatient setting;
3. Pharmaceutical representatives working for either a local company or an international company based in Lebanon.

We excluded individuals who did not speak either Arabic or English, the two languages used for consenting and interviewing.

### Recruitment and sampling

We conducted the interviews during the months of January and February of 2016. We used the known sponsor, purposive, and snowballing sampling strategies to identify interviewees from the three respective groups: physicians, pharmacists, and pharmaceutical representatives. We also aimed for maximum variation [16], by trying to recruit participants that varied by area, age, gender, and specialty.

We recruited physicians using the known sponsor approach. Each of the medical students involved in this study identified and invited, either in person, by email or by phone, an eligible physician already known to her/him. To avoid any undue influence, another team member conducted the interview.

We recruited pharmacists using a purposive sampling approach. A team member approached the staff of large pharmacies located in five major cities in Lebanon and invited them to participate. Those who agreed were interviewed by the same team member.

As for pharmaceutical representatives, one medical student identified a representative already known to her. This pharmaceutical representative identified and introduced us to other representatives. Using this snowballing strategy, we recruited the other representatives to participate in our study.

It is noteworthy to indicate that no dyad of interviewer and interviewee had previous relationships and no one approached refused to participate. We proceeded with recruitment until no new themes emerged (thematic saturation) [16].

### Data collection

We conducted the interviews in private locations convenient to the interviewees. We audiotaped the interview when permitted by the participant, otherwise, we resorted to note taking. The interviews were conducted in either English or Arabic, based on the interviewee’s preference.

First, the interviewers administered a short questionnaire asking for demographic characteristics, and then interviewed the participant using a semi-structured interview guide (S1 Appendix). Questions addressed the nature and intensity of interactions and whether these interactions are impacting drug related practices. As a way to encourage participants to be more forthcoming with information, we asked them to describe interactions in general and not necessarily their own experience. We pilot tested the first interview with each professional to ensure clarity and relevance of questions. The interviews lasted approximately 30 minutes.

### Data analysis

We did not include the data collected during pilot testing in the final analysis. We transcribed audio-recorded interviews conducted in English; while we first translated, interviews conducted in Arabic into English and then transcribed them. We anonymized all transcripts. Immediately following each interview, we proceeded with transcription, translation, and data analysis.

Two investigators worked independently on coding the transcripts line by line while maintaining a log of emerging codes, their definition, and exemplary quotes (open coding). Then they met to share, compare and contrast their independent data analyses (constant comparative technique). This debriefing helped achieve consensus on emerging codes, expand or merge codes (axial coding). A third investigator resolved disagreements. Following each discussion, we refined the interview guide to allow probing, amplifying or clarifying issues still uncovered. Investigators met to discuss the final findings and combine the themes into a conceptual framework (selective coding) [16].

### Increasing rigor

While the two senior team members (GHA and EA) had experience in conducting and publishing qualitative studies, the remaining six members (RH, AB, MC, ST, RN, FH) had no such previous experience. Therefore, we conducted training on interviewing techniques. Also, with the first few interviews, we implemented peer observation and held debriefing sessions to reflect on the process and how it could be improved.

The team members made every effort to maintain a level of reflexivity to avoid the influence of personal views and biases during data collection and data analysis, and to prevent bias during the writing stage of the study.

### Ethics

We obtained the approval of the Ethical Review Committee at the American University of Beirut prior to the initiation of the study. We obtained verbal consent from all participants. We anonymized all transcribed interviews and kept sensitive data in a separate document.

## Results

### Demographics

We recruited 32 participants: 13 physicians, 13 pharmacists, and 6 pharmaceutical company representatives. Table 1 shows their characteristics. Participants were from four different regions (Bekaa, Beirut, Mount Lebanon and South) capturing urban, suburban and rural areas in Lebanon. The majority of participating pharmacists owned a pharmacy. Half of the pharmaceutical company representatives worked in international companies. The median number of years of experience was 15, 20, and 1 for physicians, pharmacists, and pharmaceutical company representatives respectively.

**Table 1:** Characteristics of participants.

Characteristics	Physicians (N = 13)	Pharmacists (N = 13)	Pharmaceutical representatives (N = 6)
	n (%)	n (%)	n (%)
**Gender**			
Females	5 (38)	6 (46)	6 (100)
Males	8 (62)	7 (54)	—
Median years in practice (IQR)^*^	15 (12–22)	20 (7–35)	1 (1–2)
**Country of training**			
Lebanon	5 (38)	8 (62)	4 (66)
Europe	2 (16)	1 (8)	1 (17)
North America	5 (38)	2 (15)	1 (17)
Other**	1 (8)	2 (15)	—
**Location of practice**			
Beirut	5 (38)	5 (38)	4 (67)
Mount Lebanon	2 (16)	3 (23)	—
Bekaa	5 (38)	3 (23)	—
South	1 (8)	2 (16)	2 (34)
**Practice setting**			
Hospital	4 (31)	—	—
Community	6 (46)	12 (92)	—
Both	3 (23)	1 (8)	—
**Type of pharmaceutical company**			
International	—	—	3 (50)
Local	—	—	3 (50)

*IQR = Interquartile range

**Other = Physicians (Syria = 2, Libya = 1, Russia = 1); Pharmacists (Syria = 1, Ukraine = 1)

### Emerging themes

The following themes emerged from the data: purpose and driver for the interactions, nature of the interactions, incentives, impact on prescription practices, ethical considerations, and suggestions for managing the interactions. When similar views were voiced we used the term “most participants”; when that was not the case, we used the terms “many” or few” as relevant. When including quotes along with our findings, we referred to the type of participants using the following abbreviations: Physicians (*Phys)*, Pharmacists *(Pharm)*, pharmaceutical representatives *(PR)*.

### Purposes and drivers of the interactions

The main purposes of the interactions were promotional and educational as indicated by most professionals: *“Every time it’s for a different purpose*, *sometimes to invite me to a conference*, *sometimes to give me samples*, *sometimes to talk to me about the drug*.*” (Phys*_*04*_*)*. However, monitoring physicians’ drug prescription practices was another essential purpose as revealed by the three professional groups. As a means of monitoring, some pharmaceutical representatives reported that pharmacists provided them with information on physicians’ prescription of their company’s drugs, and of drugs from competitor companies: *“If you are close to the pharmacist he might tell you the name of the doctors who are prescribing or stopped prescribing or prescribing competitor products” (PR*_*01*_*)*. Others directly asked physicians for an account of the number of prescriptions for the promoted drug. Some pharmacists indicated providing representatives with access to copies of physicians’ prescriptions. This practice was justified as being a sense of obligation by many physicians or pharmacists: *“If the company that is paying money wants to know how many prescriptions a physician wrote*, *you tell them the number and some reps take a picture of the prescription to know if the physician is prescribing the drug” (Pharm*_*12*_*)*.

Most pharmaceutical representatives said that market potential was the main driver to approach certain physicians and pharmacists and not others. They target physicians who are most likely to prescribe their drugs and those with a high turnover of clients. In addition, the socio-economic status of patient population covered by physicians and pharmacists dictated the type of drug being promoted: brand names with affluent communities, and generics with less affluent communities: *“It depends on the location of the pharmacy; if it is a high-end*, *then people can afford to buy an expensive drug and so we tend to accept brand offers*. *If the region is low-end*, *we accept generics*, *same effectiveness but cheaper” (Pharm*_*04*_*)*.

### Nature of the interactions

All physicians and pharmacists’ participants had previous interactions with pharmaceutical representatives. Most of them indicated that pharmaceutical representatives’ visits are unscheduled. Very few physicians indicated having a set fixed times for visiting: “*On a daily basis […] because I am here most of the day I don’t schedule and just see them when I am free” (Phys*_*01*_*)*.

The main discussion during these interactions focused on either sharing new information about a certain new drug: “*They bring you studies*, *they ask you if you have any questions so that they can get you the answers” (Phys*_*03*_*)*, or providing evidence-based data on the benefits of their promoted drug compared to a competitor drug: *“They show you studies*, *or head to head comparisons between 2 drugs*, *sometimes some of them they try to convince you based on the financial aspect*, *for example they would tell you that this medicine is cheaper” (Phys*_*09*_*)*. However, some indicated that these interactions were of a social nature: *“Once you sit down with her and she asks about your son and tells you about hers” (Phys*_*11*_*)*.

When asked how they perceive the scientific merit of the information provided during these interactions, many physicians and pharmacists indicated that it is evidence-based. However, some suggested that the information is biased because it emphasizes the benefits of the drug while neglecting its side effects: *“If there is a study that does not show the importance of that drug or if the results were different from what they were expecting*, *they may not discuss it with us*. *But be sure that the rival drug company will discuss it with us*.*” (Phys*_*03*_*)*.

When we asked physicians and pharmacists about their perceptions of the pharmaceutical representatives’ role, some believed that they deserve sympathy: “*We should be considerate with a med rep because he is working all day long and I believe it is his utter right to know if the doctor he is dealing with is actually prescribing this company’s drug or not “(Pharm*_*04*_*)*. Others perceived them as a nuisance and at times disrespectful: *“[they] are always following physicians around and hovering in the hallways” (Phys*_*05*_*)*.

When asked how to best characterize the nature of the relationship between pharmaceutical representatives, physicians and pharmacists, most agreed that since the main purpose of this relation is to benefit patients, then there is no conflict of interest: “*If it is a medicine that you are used to prescribing a lot and it is good I don’t consider it wrong if they offer conferences or journal reviews or things of the sort*.*” (Phys*_*07*_*)*. However, the statements by many physicians and pharmacists revealed personal and financial conflict of interests: *“Each pharmacist*, *wants to profit the most of his work*, *or else no matter how professional he may be*, *if he’s not a good businessman*, *he’s not going to stay in business*.*” (Pharm*_*03*_*)*. And when not harmful to patients, some physicians reported they would modify their practices in order to maximize the benefits: *In general if there are similar medication and they are all good*, *I try to use everything*.*” (Phys*_*08*_*)*.

A few physicians admitted that the conflict of interest resulting from this relationship may be putting their patients in harm’s way “*He’ll maybe prescribe drugs that are more expensive and this will harm the patient” (Phys*_*11*_*)*. This view was seconded by one pharmaceutical representative: “Some doctors prescribe the same medication from two companies to keep receiving their deals, for example, two anti-inflammatory medications with different brand names, one pill in the morning and one pill at night” (PR_01_)

### Incentives

Pharmaceutical companies are prepared to offer a wide variety of incentives to pharmacists and physicians in return for more prescriptions. While they seem to be the primary initiators of gift giving, some physicians or pharmacists may be the initiators as well as voiced by one physician: *“There is a doctor that has a sign outside his office “if you don’t have a sample don’t bother coming in” (Phys*_*01*_).

It appears that the value of the incentive is linked to the prescription load. Pharmaceutical companies offer greater incentives for physicians with a larger number of prescriptions: *“Pharmaceutical companies classify physicians in three categories*, *according to which they decide on how much incentives they would provide*: *A (high turnover of patients)*, *B (highest turnover in their respective region) and C (low turnover of patients)*.*” (PR*_*04*_*)*. The incentives provided to physicians and pharmacists are both monetary and non-monetary and vary between the two professional groups. Monetary incentives include receiving office equipment, electronics and monthly payments while non-monetary incentives include invitations to be guest speakers at high profile meetings, or opportunities to appear on television. Therefore, they “*affect the popular reputation*, *patients would be impressed and say*: *Oh*! *They invited him to France to talk about this medicine” (Phys*_*05*_*)*. Some participants interpreted this as pharmaceutical companies giving prominence to selected physicians within their communities. This was supported by reports of pharmaceutical companies giving prominent roles to “their physicians” during professional meetings that they sponsor.

A few physicians and pharmaceutical representatives reported cases of offering of sexual favors (Table 2), provided either by the representatives themselves or through nightclub invitations or room service during travel. *“There are reps in a local company who sleep with the doctors for the sake of promotion” (PR*_*03*_*)*.

**Table 2:** List of incentives according to the three groups of participants.

Incentives	According to physicians	According to pharmacists	According to Pharmaceutical Representatives
Stationary	✔	✔	✔
Office equipment	✔	✔	✔
Office/ pharmacy furniture (including air conditioning, shelves etc.)	✔	✔	✔
Travel and accommodation	✔	✔	✔
Cash payments and grants	✔	✔	✔
Books and journal subscriptions	✔		✔
Sponsoring conferences, invitation to conferences and workshops	✔	✔	✔
Free Samples	✔	✔	✔
Electronic devices (Ipads, phones, laptops)	✔	✔	✔
Paid Key speaker positions At conferences	✔	✔	✔
Paid TV speaker	✔		
Research funding	✔		✔
Position as medical advisory board at the company	✔		✔
Car loans		✔	
Sexual (pay for strip clubs/prostitutes) or drug representatives engage in sexual acts	✔	✔	✔
Free meals	✔	✔	✔
Deals and discounts		✔	✔
Pay syndicate fees	✔		
Pay for marriage/ honey moon			✔
Local retreats and boat trips and family vacations	✔		✔

Pharmaceutical company representatives were also incentivized. They received bonuses when they increase the sales and revenues of their company. Conversely, poor performance provided ground for warnings and potentially dismissal. Pharmaceutical representatives also benefit from free consultations from physicians and discounted medication from pharmacists.

### Impact on prescription practices

The impact of the relationship between physicians, pharmacists and pharmaceutical representatives and its resulting conflicting interest varied by individuals. A few physicians insisted that their own prescription practices were not affected: “*I don’t prescribe it if I am not convinced*, *some of the drug reps are actually my friends and I still don’t prescribe for them if I am not convinced*.*” (Phys*_*09*_*)*. Some physicians said that when affronted by two similar drugs from competitor companies, they were inclined to prescribe the drug from the company that provided them the incentive. They indicated that it was a sense of obligation towards that company that drive their decision. *“It is so difficult to say they supported me and I am not going to prescribe for them” (Phys03)*. Many physicians, however, observed that over-prescription of unnecessary and expensive drugs are common occurrences among physicians in their communities: *“Take for example anti-osteoporosis medicines the doctor might tell people your bones are very good but this drug is preventive you should take it so they prescribe randomly” (Phys11)*.

Some physicians indicated that pharmacists tend to “*sell the more expensive drugs because they benefit more” (Phys02)*. Several pharmacists reported that some pharmacies might be dispensing medication even without prescription: *“Sometimes*, *someone would come to buy a medicine without prescription; we tell them no we can’t but they go to another pharmacy and they give it” (Pharm08)*. In addition, many pharmacists alluded to mutual referrals between physicians and pharmacists to maximize their benefits: *“The physician sends [patient]) to the pharmacy and the pharmacy gives the physician a certain commission and recommends the physician*.*” (Pharm07)*.

### Ethical considerations

There was an overall culture of acceptance of the gift giving. The boundaries between what is considered ‘ethical’ and ‘unethical’ in the gift giving were not consistent amongst participants. Some considered acceptance of gift of any kind to be unethical: “*There shouldn’t be any promotional material such as gadgets*, *travels*, *covering trips*, *sponsoring of research” (Phys*_*03*_*)*. Others believed that educational gifts or free samples for example, are acceptable provided these free samples are dispensed to patients and not sold: *‘There are some doctors who really do it (use the incentives/samples) in a good way to help unfortunate people” (Phys*_*11*_*)*. Some participants noted that some physicians sell the free samples they collect from pharmaceutical representatives: “*They [physicians] tell you that they are giving you a reduction on this medicine although they received it for free*.*” (Phys*_*10*_*)*

However, there was a consensus that anything that may harm the patient because of this interaction is unethical and should be a “red line”: “*At the end of the day the aim is financial gain*. *The red line is whenever the health of the patient is put into jeopardy”* (*Pharm*_*05*_).

### Suggestions for managing the interactions

Physicians and pharmacists indicated the need for laws to regulate interactions between physicians and pharmaceutical representatives. Some proposed adding caps to incentives “*I think there should be rules and regulations and some things should be allowed and others not allowed especially promotional material offered to the doctors it should only be limited to scientific material” (Phys*_*03*_*)*. A few physicians also proposed the prohibition of the monitoring of prescription practices by pharmaceutical companies: “*I think it is unethical for them to know who is prescribing what but to know in general what their sales are*, *they should be allowed” (Phys*_*07*_*)*. Others suggested implementing a reporting system that keeps track of physician’s prescription patterns, and their interactions with pharmaceutical companies: *“I think it is important to monitor prescription patterns*, *especially if there is an accurate reporting system like gains or gifts that physicians get from pharmaceutical companies*.*” (Phys*_*05*_*)*.

Some participants voiced skepticism about the efficacy of laws. A few indicated that in the absence of a mechanism for monitoring the interaction and prescription practices, these laws are useless: “*Since it is not computerized*, *nobody will know*, *they (authorities) cannot catch hundreds and thousands of papers of what is sold and what is done every day” (Phys*_*02*_*)*.

The problematic behaviors seem to be more common in interactions with representatives of local pharmaceutical companies, compared to representatives of international companies. Pharmaceutical representatives highlighted the need for local companies to adopt policies similar to those of international companies: *“We could follow Big international pharmaceutical companies which have two ethical centers*: *a medical department that imposes selection criteria for congresses*, *and an audit office that checks and audits all expenses and activities*. *Unlike local companies no cash advances are made by multinational pharma” (PR*_*03*_*)*.

## Discussion

**Fig 1:**
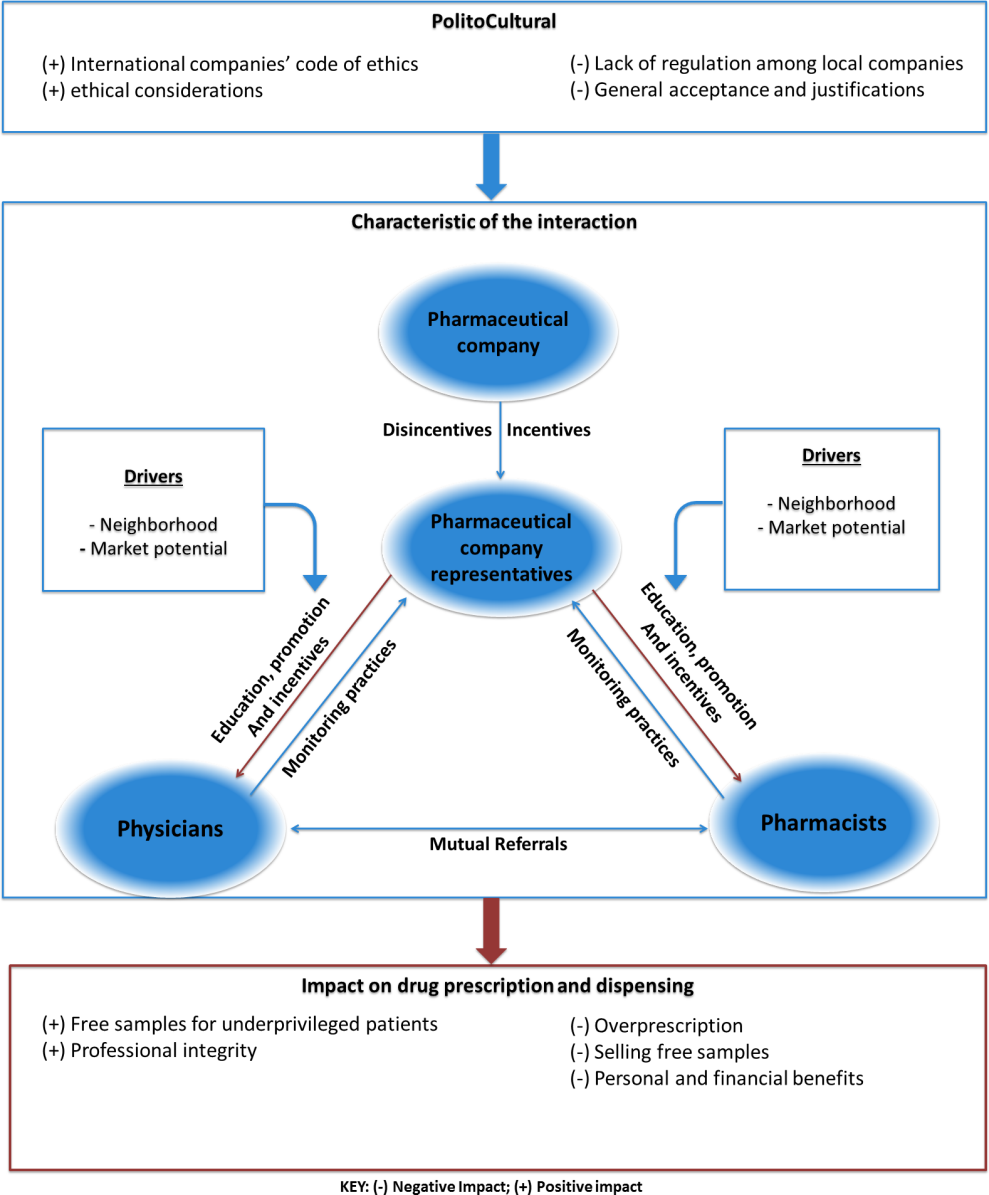
Descriptive framework. The framework depicts the interaction between physicians, pharmaceutical representatives and pharmacists, the influence of the policy and culture on this interaction and the effects of this interaction on drug prescribing and dispensing practices. (+) denotes positive influence and (-) denotes negative influence.

The three professional groups described the prevailing politico-cultural environment, the various mechanisms that characterize their interactions, and the impact of these interactions on prescription practices and dispensing. These multifaceted factors are depicted in Fig 1.

The main purposes for the interaction were educational, promotional, and monitoring prescription practices and dispensing, while the main drivers for these interactions were market potential and neighborhood socio-economic status. The heavily laden incentive marketing strategy permeated all these interactions, starting with the pharmaceutical companies rewarding (or penalizing) their representatives for reaching (or lack of) their sale targets, who in turn were providing physicians and pharmacists with a range of incentives in return of more sales. Mutual referrals between physicians and pharmacists were also used as a strategy to maximize benefits. All study participants described the influence of these interactions on drug prescription and dispensing. There was an overall acceptance of pharmaceutical representatives’ interactions with physicians and pharmacists. In some cases, the international companies’ code of ethics and the professional integrity counterweighted the absence of national policies and the lack of regulation among local companies.

Our findings appear to be similar to studies conducted in other LMIC in the region such as Jordan, Egypt and Iraq [12, 18, 19]. A study conducted in Jordan revealed a wide acceptance of gift-giving to physicians and a significant impact on prescription practices of physicians[18]. Similarly, in Egypt, a study demonstrated that physicians are aware of the pharmaceutical companies’ promotional practices and their influence on prescription practices[12]. In Iraq also there seems to be a general acceptance of gift-giving with a shift in prescription practices correlating with gift acceptance[19]

In 2001, Lakoff, a social anthropologist, examined whether the surge in antidepressant post financial crisis in Argentina was associated with the social situation or with promotional practices of the pharmaceutical industry. Lakoff concluded that the surge could be best explained by the work of sales representatives and opinion leaders to convince doctors to prescribe the newer SSRIs [20]. Lakoff had similar concerns over the ubiquity of gift giving and its impact on the ethical discourse, the “pharmaceutical audit industry” practices, and the resulting impact of the “high-contact” between pharmaceutical industry and physicians on prescription practices.

Oldani described the “gift cycle”, where medications move from pharmaceutical representatives to physicians to patients; the gifting then returns to the pharmaceutical company as revenue. Indeed, the patients will feel indebted to the physician and the company, and wanting to directly reciprocate to the pharmaceutical company, they will buy the medication. [21]

In May 2016, the Ministry of Public Health in Lebanon, and while we were conducting this study, launched the first national code of ethics to guide health professionals on how to manage their interactions with the pharmaceutical industry. This code of ethics addresses the marketing and promotion practices to be applied by both health professionals and the pharmaceutical industry based in Lebanon, covering issues such as: direct to consumer advertisement; value of promotional items allowed, cash payments, allowable quantities of free samples; sponsorship of congresses and marketing of drugs, and the disclosure by pharmacists of information related to physician’s prescribing patterns. About 20 international pharmaceutical companies and nearly 20 national pharmaceutical companies pledged and signed the code of ethics indicating their voluntary compliance to its clauses [22].

In this study, participants described various marketing strategies commonly adopted by pharmaceutical companies to promote their drugs. These include the use of personal visits to detail the benefits of the promoted drug and the use of incentives as a ‘quid pro quo’ approach to influence prescription practices. The type of incentives reported ranged from office equipment to gold coins, paying car loans, unrestricted grants and sexual favors. An article recently published in “The Guardian” investigated allegations of sexual favors and bribery by employees of an international pharmaceutical company in Jordan and Lebanon [23]. This practice raises serious ethical concerns especially when compounded by their apparent prevalence.

The International Federation of Pharmaceutical Manufacturers and Association [24] (IFPMA) clearly indicates that personal gifts should be explicitly banned but promotional aids of minimal value and relevant to professional practice may still be allowed (e.g. branded pens and pads), as are items of medical utility for patient care (e.g. textbooks and anatomical models). Similarly, the recently released code of ethics for medicinal products promotion in Lebanon prohibits gifts worth more than 10% of the monthly minimum wage [22, 25]. Future study needs to examine the impact of this new law on the gift-giving practices in Lebanon.

The monitoring of prescription practices observed in this study has been a worldwide pharmaceutical company strategy since the 1950s [25]. In the US, pharmaceutical companies purchase prescription data from pharmacists through information distribution companies [26]. Even though this information is coded, the pharmaceutical companies are capable of identifying physicians [26]. As a result, some states including Vermont, New Hampshire and Maine tried to limit the monitoring of physicians’ prescriptions [27]. For example, Vermont’s law prohibited pharmacies from selling, licensing, or exchanging prescriber identifiable prescription information and from permitting its use for drug promotion. Pharmaceutical manufacturers and marketers were likewise prohibited from using the information for marketing purposes.

Our finding that many pharmacists admitted to sharing copies of prescriptions with pharmaceutical representatives is very concerning. These practices breach the principle of confidentiality for both the prescriber and the patients. Strict regulations and governance of pharmaceutical and providers need to be established in Lebanon following the example of Estonia. In 2002, Estonia passed the Health Services Organization Act that led to the establishment of a separate stage agency to supervise private providers and ensure implementation of best practices, including those related to confidentiality [28].

The practice of selling marketing samples of medicines is also concerning. The Lebanese code of ethics indicates that pharmaceutical representatives could provide doctors with small quantities of samples “in packages not intended for sale”, “to introduce them to the product”. However, our study was conducted prior to the announcement of the code of ethics in May 2016. Further research is needed to evaluate whether this new code of ethics has limited this practice. Other countries, for example those who are part of the IFPMA, have enacted laws to regulate the use of drug samples in marketing [24].

Globally there are control systems that govern the interactions between pharmaceutical companies and health care providers including: international and national industry codes of practice; internal company standards; and national laws and regulations [25]. These control systems are intended to supply health care providers with the best available evidence needed for informed decision-making and to uphold ethical practices in communication [25]. For example, the IFPMA is responsible for developing standards of what companies can claim about their products and the interactions their employees can have with health care professionals[24]. Multinational pharmaceutical company members of IFPMA are bound to abide by those standards worldwide. The national codes of practice are those issued to control advertising and interaction and usually apply to local national market and beyond, where these companies have a market (e.g. Association of the British Pharmaceutical Industry) [29]. Internal company standards are the self-regulation system of individual pharmaceutical companies, these are reinforced by audit organizations. Local laws and regulations, are meant to deter improper activities through enforcement and judicial measures. For example, in Europe, if an inappropriate payment or gift is given or offered by a company or requested or accepted by a healthcare professional, both parties could be penalized [25].

Where does Lebanon fare in terms of these control systems? International pharmaceutical companies promoting their drugs in Lebanon are bound to the terms and regulations of IFPMA. Hence, they are subject to codes and legislations as pointed out by participants in our study. While the ministry of public health in Lebanon released a national code of practice entitled: “Code of ethics for medicinal products promotion in Lebanon and implementation procedures”[22], it has not put in place the needed processes for its implementation. Therefore, domestic pharmaceutical companies may not be routinely subject to any robust controls on their advertising and related activities, and there is little information about the internal company standards regarding promotion and interaction practices. In view of these gaps, several control mechanisms need to be introduced into the Lebanese health care system including the implementation of the Code of ethics for medicinal products promotion in Lebanon and implementation procedures and encouraging domestic pharmaceutical companies to become members of the IPFMA.

## Strengths and limitations

To our knowledge, this is the first study to qualitatively explore the interactions between physicians, pharmacists and pharmaceutical representatives in the Middle East. Major strengths of the study include (1) the triangulation approach by including participants from the three groups of interest; (2) the expertise amongst the team members in the subject matter as well as in qualitative methods; (3) the thorough training of investigators in both data collection and data analysis; (4) conducting the analysis in a duplicate and independent manner followed by consensus building.

Due to the sensitivity of the topic, there is a risk that participants were not completely forthcoming in answering our questions leading to some underreporting. However, the inclusion of the three groups of interest should have minimized this risk. Another common limitation of qualitative studies is the transferability of findings to other settings, but we suspect that many of our findings apply to other contexts, particularly those in other middle-income countries.

### Implications

Physicians, as individuals as well as a body, need to lead efforts to minimize any real or perceived unprofessional or unethical interactions with pharmaceutical representatives. They also need to minimize any influence of interactions deemed as acceptable on their prescription behavior. The government can also have a major role through forming an independent agency, whose role include supervision of all providers; monitoring of physician prescription similarly to what is done in Canada (Prescription Monitoring Program www.nspmp.ca); and establishing an independent advisory committee to reinforce and monitor the implementation of the code of ethics. Similarly, there is a need to identify alternative unbiased sources for continuous medical education such as practice guidelines, and peer-reviewed research. The government also needs to enhance the implementation of recently launched initiatives such as the generic drug substitution policy[30].

Future research needs to quantify the extent to which the different types of interactions and incentives are prevalent among physicians in Lebanon. It would also be interesting to explore whether the findings of this study are reproducible in other settings, particularly other LMICs. Another relevant area of research is the public as well as patients’ awareness and perceptions of interactions, and how they affect their confidence in the healthcare system.

## Conclusion

In this study, we provided a conceptual framework that describes the prevailing politico-cultural environment, the interactions between physicians, pharmacists, and pharmaceutical company representatives, and the impact of these interactions on prescription and dispensing practices in Lebanon. We found that the interactions between physicians, pharmacists and pharmaceutical representatives are frequent. While these interactions may be beneficial, they can negatively impact drug prescription and dispensing practices.

## Supporting information

S1 Appendix Interview guide. (PDF)

S1 File COREQ checklist. (DOC)
