# Supplementary material for: Characterizing the interaction between physicians, pharmacists and pharmaceutical representatives in a middle-income country: A qualitative study
Source: PLoS One. 2017 Sep 12;12(9):e0184662. doi: 10.1371/journal.pone.0184662 (PMC5595332; doi:10.1371/journal.pone.0184662)
Supplement: S1 Appendix — (PDF) [file pone.0184662.s001.pdf]

1. Interview with Physicians:

Introduction of project and interviewer:

Hello, my name is ______, I am currently a medical student at the American University of Beirut. We are conducting a research study and I would like to ask you some questions concerning the interaction between physicians and pharmaceutical company representatives within the scope of our research topic regarding the interaction between pharmaceutical companies, physicians, and pharmacists.

Nature and intensity of interaction:

Main questions	Additional questions	Clarifying questions
1- How do physicians interact with pharmaceutical companies?<br><br>2- Do pharmaceutical company representatives visit physicians? How frequently?<br><br>3- What is the aim of the visit?<br><br>4- Why is it important for a physician to be visited by pharmaceutical company representatives?<br><br>5- Does what the pharmaceutical companies offer depend on the type of the drug?	1- Daily? Weekly? Monthly?<br><br>2- Do physicians accept all visits? Or do they have any specific criteria?<br><br>3- Is the aim educational or promotional?<br><br>4- Do physicians form personal relations with pharmaceutical company representatives or is it purely professional?<br><br>5- Do physicians offer free consultations to the pharmaceutical company representatives based on the relationship they form with them?<br><br>6- Do physicians receive any of the following:<br>● Information about the new drugs hitting the	1- Can you clarify on this?<br><br>2- Can you tell me anything else?<br><br>3- Can you give an example?

	medical field?<br>● Free drug samples<br>● Invitations to conferences or workshops?<br>● Pens, notepads, …<br>● Free meals, lunches, or dinners?<br>● Books?<br>● Educational benefits (i.e. do physicians get to continue their studies further via the help of these companies)<br>● Financial benefits?<br>● Funding research projects?<br>● Luxurious gifts: travels, phones, etc.?	

Compliance:

Main questions	Additional questions	Clarifying questions
1- How often do physicians prescribe the drug after the pharmaceutical company representatives visit them?<br><br>2- Would physicians prescribe the drug more often, after a pharmaceutical company representative visits them?	1- Do physicians have to prescribe a certain amount of drugs once they form a relation with the pharmaceutical company?	1- Can you clarify on this?<br><br>2- Can you tell me anything else?<br><br>3- Can you give an example?

Monitoring:

Main questions	Additional questions	Clarifying questions
1- Do pharmaceutical companies monitor physicians' prescriptions?<br><br>2- Are you aware of the methods these companies use to monitor?	1- Do pharmaceutical companies have access to physicians' prescriptions?<br><br>2- Are you aware of a relation between the pharmaceutical company representatives and pharmacies and the possibility of using this relation as a mean of monitoring the prescriptions?	1- Can you clarify on this?<br><br>2- Can you tell me anything else?<br><br>3- Can you give an example?

2. Interview with of pharmaceutical company representatives who visit physicians:

Introduction of project and interviewer:

Hello, my name is ______, I am currently a medical student at the American University of Beirut. We are conducting a research study and I would like to ask you some questions concerning the interaction between pharmaceutical company representatives and physicians within the scope of our topic regarding the interaction between pharmaceutical companies, physicians, and pharmacists.

Nature and intensity of interaction:

Main questions	Additional questions	Clarifying questions

1- Do you visit physicians? How often?<br><br>2- What is the aim of the visit?<br><br>3- Why is it important for you to visit physicians?<br><br>4- How do you convince physicians to prescribe your product?<br><br>5- Does what the pharmaceutical companies offer depend on the type of the drug?	Daily? Weekly? Monthly?<br><br>Where do you meet with physicians?<br><br>Based on what criteria do you chose the physicians you visit?<br><br>Is the aim educational or promotional?<br><br>Do you form personal relations with physicians or is it purely professional?<br><br>Do you give them scientific proves about the effect of the product?<br><br>Do you invite them to attend seminars/ lectures explaining the benefits of the drug?<br><br>Do you offer the physicians any of the following:<br>● Information about the new drugs hitting the medical field?<br>● Free drug samples<br>● Invitations to conferences or workshops?<br>● Pens, notepads, …<br>● Free meals, lunches, or dinners?<br>● Books?<br>● Educational benefits (continue their education at your expense)<br>● Financial benefits?	1- Can you clarify on this?<br><br>2- Can you tell me anything else?<br><br>3- Can you give an example?

	• Funding research projects<br>• Luxurious gifts: travels, phones, etc.?	

Monitoring:

Main questions	Additional questions	Clarifying questions
1. How do you make sure that the physicians are prescribing your product?<br>2. Do you have any specific methods of monitoring?	1. Do you pay surprise visits to physicians' clinics to make sure they are prescribing your product?<br>2. Do you have access to physicians' prescriptions?<br>3. Do you work with pharmacies to monitor the drug prescribed by each physician?	1. Can you clarify on this?<br>2. Can you tell me anything else?<br>3. Can you give an example?

3. Interview with pharmaceutical company representatives who visit pharmacists:

Introduction of project and interviewer:

Hello, my name is _____, I am currently a medical student at the American University of Beirut. We are conducting a research study and I would like to ask you some questions concerning the interaction between pharmaceutical company representatives and pharmacists within the scope of our topic regarding the interaction between pharmaceutical companies, physicians, and pharmacists.

Nature and intensity of interaction:

Main questions	Additional questions	Clarifying questions
1- Do you visit pharmacists? How often?<br><br>2- What is the aim of the visit?<br><br>3- Why is it important for you to visit pharmacists?<br><br>4- Do you promote and convince pharmacists to suggest your products if they are available over the counter?<br><br>5- Does what the drug companies offer depend on the type of the drug?	1- Daily? Weekly? Monthly?<br><br>2- Where do you meet with pharmacists?<br><br>3- Do you use this relationship as a mean to monitor the physicians?<br><br>4- Based on what criteria do you chose the pharmacists you visit (i.e. distance from the physicians you meet, the status of the pharmacy)?<br><br>5- Is the aim educational or promotional?<br><br>6- Do you form personal relations with pharmacists or is it purely professional?<br><br>7- Does your encounter with the pharmacists help you in monitoring physicians' prescriptions?<br><br>8- Do you have access to pharmacy files?	1- Can you clarify on this?<br><br>2- Can you tell me anything else?<br><br>3- Can you give an example?

	9- Do you offer pharmacists any of these items:<br>● Free drug samples<br>● Pens, notepads, …<br>● Free meals, lunches, or dinners?<br>● Books?<br>● Others?	

4. Interview with Pharmacists:

Introduction of project and interviewer:

Hello, my name is ______, I am currently a medical student at the American University of Beirut. We are conducting a research study and I would like to ask you some questions concerning the

interaction between pharmacists and pharmaceutical company representatives within the scope of our topic regarding the interaction between pharmaceutical companies and physicians and pharmacists.

Nature and intensity of interaction:

Main questions	Additional questions	Clarifying questions
1. Do pharmaceutical company representatives visit you? How often?<br>2. What is the aim of the visit?<br>3. Why are you willing to meet and help pharmaceutical company representatives?<br>4. What are pharmaceutical companies willing to provide pharmacies with for the sake of information?<br>5. Do pharmacists prescribe drugs over the counter? Do pharmaceutical companies promote such actions?<br>6. Does what the pharmaceutical companies offer depend on the type of the drug?	1. Daily? Weekly? Monthly?<br>2. Where do you meet with the drug representatives?<br>3. Is the aim educational or promotional?<br>4. Do pharmaceutical company representatives use this relationship as a mean to monitor physicians' prescriptions?<br>5. Do you form personal relations with pharmaceutical company representatives or is it purely professional?<br>6. Do you provide pharmaceutical company representatives with discounts on drugs based on the relationship that developed between you?<br>7. Do pharmaceutical companies provide you with:<br>● Information about the new drugs hitting the medical field?	1. Can you clarify on this?<br>2. Can you tell me anything else?<br>3. Can you give an example?

	• Free drug samples?<br>• Tempting offers?<br>• Invitations to conferences or workshops?<br>• Pens, notepads, …<br>• Free meals, lunches, or dinners?<br>• Books?<br>• Educational benefits (i.e. do you get to continue your studies further via the help of these companies)<br>• Financial benefits?<br>• Luxurious gifts: travels, phones, etc.?	

Compliance:

Main questions	Additional questions	Clarifying questions
1- How often do you comply with the pharmaceutical company representatives' requests?	1- If pharmaceutical company representatives find out that the physician they are monitoring is not prescribing the drug they are promoting, what is their possible reaction?	1- Can you clarify on this?<br><br>2- Can you tell me anything else?<br><br>3- Can you give an example?

Monitoring:

Main questions	Additional questions	Clarifying questions

1. Is this relation used as a means of monitoring physicians' prescriptions?	1- Do pharmaceutical company representatives ask for your help to monitor physicians' prescriptions?<br><br>2- To what extent are you willing to interact with pharmaceutical company representatives?<br><br>3- Do pharmaceutical companies have access to physicians' prescriptions i.e. do they ask for physician's prescriptions?	1- Can you clarify on this?<br><br>2- Can you tell me anything else?<br><br>3- Can you give an example?
